# Testing Memories of Personally Experienced Events: The Testing Effect Seems Not to Persist in Autobiographical Memory

**DOI:** 10.3389/fpsyg.2018.00810

**Published:** 2018-05-24

**Authors:** Kathrin J. Emmerdinger, Christof Kuhbandner

**Affiliations:** Department of Psychology, Faculty of Psychology, Education, and Sport Science, University of Regensburg, Regensburg, Germany

**Keywords:** testing effect, retrieval practice, autobiographical memory, personal memory, long-term memory, hypermnesia, emotional memory

## Abstract

Numerous studies have shown that retrieving contents from memory in a test improves long-term retention for those contents, even when compared to restudying (i.e., the “testing effect”). The beneficial effect of retrieval practice has been demonstrated for many different types of memory representations; however, one particularly important memory system has not been addressed in previous testing effect research: autobiographical memory. The aim of the present study was to examine the effect of retrieving memories for personally experienced events on long-term memory for those events. In an initial elicitation session, participants described memories for personally experienced events in response to a variety of cue words. In a retrieval practice/restudy session the following day, they repeatedly practiced retrieval for half of their memories by recalling and writing down the previously described events; the other half of memories was restudied by rereading and copying the event descriptions. Long-term retention of all previously collected memories was assessed at two different retention intervals (2 weeks and 13 weeks). In the retrieval practice session, a hypermnesic effect emerged, with memory performance increasing across the practice cycles. Long-term memory performance significantly dropped from the 2-weeks to the 13-weeks retention interval, but no significant difference in memory performance was observed between previously repeatedly retrieved and previously repeatedly restudied memories. Thus, in autobiographical memory, retrieval practice seems to be no more beneficial for long-term retention than repeated re-exposure.

## Introduction

Numerous studies have shown that retrieving contents from memory in a test considerably improves long-term memory for those contents, even when compared to a condition where the contents are represented for restudying (i.e., the “testing effect,” Carrier and Pashler, 1992; for recent meta-analyses see Rowland, 2014; Adesope et al., 2017). The beneficial effect of retrieval practice has been demonstrated for a wide range of test formats (e.g., cued recall, free recall, recognition memory), for a large variety of study materials (e.g., wordlists, vocabulary, prose texts), and even extends to procedural skills (Kromann et al., 2009) and emotional memories(Emmerdinger et al., 2017). However, previous research in the field has neglected one particularly important memory system: autobiographical memory. While a few studies have examined the effect of retrieving autobiographical events from memory compared to non-retrieved autobiographical events (Barnier et al., 2004; Stone et al., 2013), to our knowledge, no study to date has examined the effect of testing autobiographical memories compared to repeated re-exposure to those memories.

Autobiographical memories, the recollections of personally experienced events, are a unique type of memory representations characterized by a high degree of complexity and a strong interconnectedness in an associative network. Autobiographical memories are thought to be hierarchically organized, such that detailed recollections of specific events are embedded in a rich context of more abstract knowledge about the personal past (Conway and Pleydell-Pearce, 2000). Importantly, autobiographical memories are also highly linked to the self, thus interrelated with personal motives and evaluations, and often emotionally significant (Conway, 2005).

It is an open question if the retrieval benefits reliably observed for non-personal learning materials persist for personal, self-related information. Generally, it has been shown that previously retrieved autobiographical events are better retained than non-retrieved autobiographical events (Barnier et al., 2004; Stone et al., 2013). However, less is known regarding the relative significance of retrieving personally experienced events compared to re-exposure to those events, as may occur for example when listening to rehearsals of socially shared personally experienced events in conversations. Interestingly, there is some evidence that retrieving socially shared personally experienced events in the role of the speaker benefits memory retention similarly to being re-exposed to those events in the role of the listener (Stone et al., 2013). This finding may hint at the possibility that retrieval practice may not necessarily benefit long-term memory for personally experienced events more than repeated re-exposure to those events.

Indeed, based on theoretical accounts of why retrieving information in a test benefits long-term memory, one may speculate that the testing effect may be less pronounced or even disappear for autobiographical memories of personally experienced events. Existing explanations for the testing effect rest upon the assumption that the successful retrieval of a memory initiates elaborative processes that update and strengthen the memory trace through the establishment of new relations (Carpenter, 2009, 2011; Pyc and Rawson, 2010; Kornell et al., 2015). However, this benefit received from testing may be reduced or even absent for autobiographical memories, which typically exhibit inherently strong links in an associative network. In fact, recent studies failed to replicate the testing effect for complex, highly associated materials (De Jonge et al., 2015; van Gog et al., 2015; for a review, see van Gog and Sweller, 2015). For example, in the study of De Jonge et al. (2015), while taking a test on a previously studied strongly interrelated text did not benefit memory any more than restudying the text, the typical retrieval benefit did emerge when the same text was scrambled and presented for studying and retrieval practice as single, non-related facts. On the other hand, a simple re-exposure to the material, as typically realized in the restudy condition, may benefit memory for personally relevant information more than memory for non-personal information. Numerous studies have shown increased memory performance for information that is relevant to the self (for a review, see Symons and Johnson, 1997). Due to the highly associatively organized and self-related nature of autobiographical memory representations, repeated re-exposure alone may lead to similar memory benefits than actively retrieving the information, and in consequence, attenuate the typical pattern of the testing effect. Following a distribution-based perspective (Kornell et al., 2011), the testing effect may thus emerge, if at all, only after comparably long retention intervals, because not only previously successfully retrieved, but also restudied memory representations would stay rather long well above the recall threshold.

In the current study, we extended the testing effect paradigm to autobiographical memories. For this purpose, we adapted an experimental procedure developed by Barnier et al. (2004) using autobiographical memories for personally experienced events elicited in response to cue words. The present experiment consisted of four sessions: An initial collection session, a retrieval practice/restudy session the following day, and two delayed memory test sessions at different retention intervals (2 weeks and 13 weeks). In the collection session, participants were asked to describe events they had personally experienced in the last 6 months in response to a variety of cue words. To control for possible effects of emotional significance, one third of the cue words was emotionally negative, one third neutral, and the remaining third positive. The retrieval practice/restudy session took place 1 day after the collection session. Each participant was provided with the descriptions of half of his or her personal events of each emotional quality together with the corresponding cue for restudy. Participants were asked to carefully read the cue word and the event description and copy the event description by hand. For the other half of the events, only the cues were presented, and participants were asked to retrieve and write down the corresponding memories. After 2 weeks, we assessed delayed memory performance for all originally collected autobiographical events; participants were asked to recall and write down all originally described events in response to the corresponding cue words. To control for the possibility that for autobiographical material the benefits of retrieval practice might only emerge after a comparably long retention interval, the delayed memory test was repeated about 3 months after the retrieval practice/restudy session.

## Materials and Methods

### Participants

A power analysis was performed for sample size estimation and revealed that to achieve a power of 0.80 for detecting small to medium sized effects (*d* = 0.4, α = 0.05; G^∗^Power 3.1.7; Faul et al., 2007), a sample size of at least 41 would be required. Thus, we decided to recruit 48 undergraduate students (45 females, *M*_Age_ = 21.3, *SD* = 5.5) who participated for course credit. The study was approved by the Ethics Committee of the University of Regensburg. All participants provided written informed consent in accordance with the Declaration of Helsinki.

### Materials

In an initial collection session, participants were asked to describe personally experienced events from the last 6 month in response to cue words. For this purpose, three cue word lists were constructed that contained either 36 neutral (e.g., shoe, table, buy), 36 emotionally positive (e.g., entertaining, friendship, happiness), or 36 emotionally negative (e.g., sick, quarrel, lonely) German words (for the complete cue word lists in German and their English translations, see Supplementary Table 1). Cue words were selected from previous studies using the cue word method for the collection of autobiographical memories (Robinson, 1976; Maccallum et al., 2000; Barnier et al., 2004, 2007; Kuyken and Dalgleish, 2011), and translated into German, and from the Berlin Affective Words List Reloaded (Võ et al., 2009), a list of affective German words.

We slightly modified the collection procedure applied by Barnier et al. (2004) who had instructed participants to describe events for each of the presented cue words, and allowed participants to choose individually from a broader selection of cue words in order to assure that participants were able to describe a sufficient sample of authentic autobiographical events. Participants received the neutral, positive, and negative cue word lists and a booklet to write down their autobiographical events and to rate them according to a number of characteristics (see below). For each cue word list, participants were asked to describe a specific event they had personally experienced within the last 6 months in response to 16 cue words, and to write down a description of the event in one or two sentences. Participants were told not to describe routines (e.g., “Thursdays I usually go swimming”) but specific events. They were instructed to think of non-emotionally significant events in response to the neutral cue words, and of emotionally positive or emotionally negative events in response to the emotionally positive or negative cue words. To prevent ceiling effects due to divergent cognitive processing of extremely emotionally charged events (e.g., Brown and Kulik, 1977; Wagenaar and Groeneweg, 1990; for a review see Christianson and Safer, 1996), for emotionally positive and negative cue words, participants were asked to think of medium intense emotionally positive and negative events in their everyday life, rather than of profound, drastic events.

Participants worked successively on all three cue word lists; the order in which the neutral, positive, and negative cue word lists were provided was counterbalanced across participants. Immediately after having written down the description of an event, participants rated the event on 7-point-scales according to its clarity (“How clear is your memory of the event?”; 1 = not clear at all, 7 = very clear), personal relevance (“How personally relevant/significant is this memory for you?”; 1 = not relevant at all, 7 = very relevant), emotional valence (“How positive or negative is this memory for you?”; 1 = very negative, 7 = very positive), emotional arousal (“How emotionally arousing is this memory for you?”; 1 = not at all emotionally arousing, 7 = very emotionally arousing), and the frequency with which they had previously thought of this event or told others about it (“How often did you think about this memory or told others about it?”; 1 = not at all, 7 = very often). **Table 1** shows the characteristics of the collected sample of autobiographical events (for a supplemental analysis of ratings of memory characteristics as a function of emotion condition and assigned practice condition, see Supplementary Data Sheet 1).

**Table 1:** Mean ratings for the characteristics of the sample of autobiographical memories retrieved by participants in response to neutral, positive, and negative cue words.

	Emotional quality of cue word					
	Neutral		Positive		Negative	
Ratings of memory characteristics	*M*	*SD*	*M*	*SD*	*M*	*SD*
Clarity	5.07	0.98	5.98	0.58	5.76	0.48
Personal relevance	2.51	0.91	4.83	0.88	4.43	0.92
Emotional valence	4.07	0.25	6.01	0.47	2.25	0.40
Emotional arousal	2.23	1.02	4.71	0.83	4.80	0.83
Frequency of previous retrieval	1.99	0.66	3.99	0.91	3.86	0.90

*Participants rated their memories in terms of clarity (1 = not clear at all, 7 = very clear), personal relevance (1 = not relevant at all, 7 = very relevant), emotional valence (1 = very negative, 7 = very positive), emotional arousal (1 = not at all emotionally arousing, 7 = very emotionally arousing), and the frequency with which they had previously thought of this event or told others about it (1 = not at all, 7 = very often).*

### Design and Procedure

**FIGURE 1:**
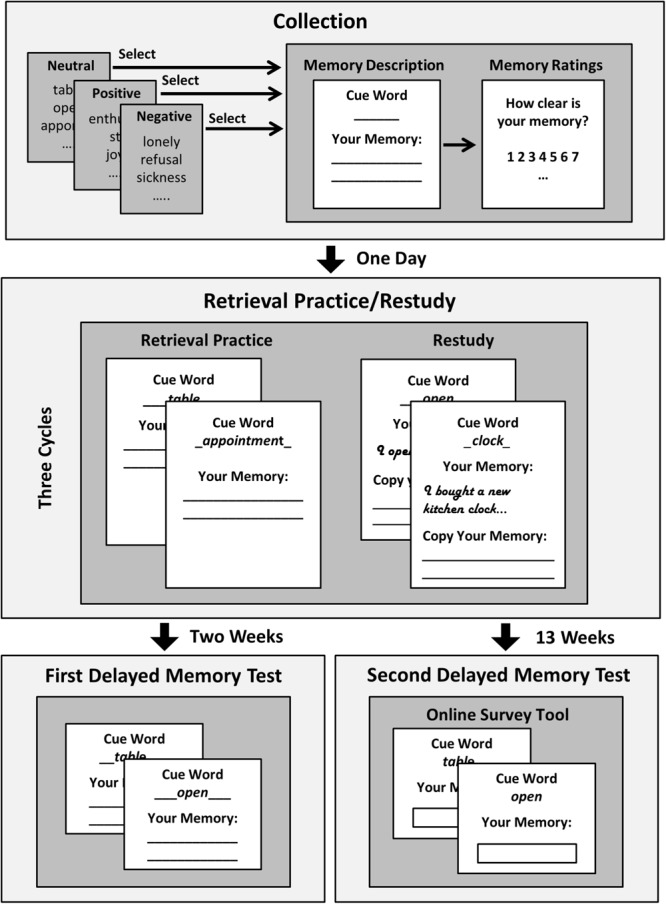
Procedure of the experiment. In an initial collection session, participants described 48 autobiographical events (16 neutral, 16 positive, and 16 negative) in response to cue words. In a retrieval practice/restudy session the following day, for half of the collected events, participants received only the cue words, and they were asked to remember and write down the corresponding event (retrieval practice); for the other half of the events, the cue words were presented together with the collected descriptions, and they were asked to restudy and copy the descriptions (restudy). Overall, participants completed three retrieval practice/restudy cycles. In two delayed memory test sessions (2 weeks and 13 weeks after the retrieval practice/restudy session), memory for all 48 originally collected autobiographical events were tested (for details, see section “Materials and Methods”).

**Figure 1** depicts the procedure of the experiment. The experiment consisted of four sessions. An initial collection session, a retrieval practice/restudy session the following day, and two delayed memory test sessions that took place at different retention intervals (2 weeks and 13 weeks after the retrieval practice/restudy session). The first three sessions took place in the laboratory and the second delayed memory test after 13 weeks was conducted via an online survey tool (SoSci Survey; Leiner, 2014) where participants accessed their individualized tests with a code.

#### Collection Session

At the beginning of the initial collection session, participants were told that they were taking part in an experiment investigating how people remember and cope with autobiographical memories of emotional and non-emotional events, and that they would be asked to describe personally experienced events in the present session, and work in different ways on their memories about these events in the following sessions. They were informed that the experimenter would have no access to the booklets containing their event descriptions, but that independent research assistants would collect and code their anonymized booklets, thus ensuring that none of the collected events would be assignable to any individual person. Participants were instructed that they would subsequently work on three cue word lists and that, for each list, their task consisted in sequentially selecting a total of 16 cue words and describe personally experienced events from the last 6 months in response to them. They were told that there was a list with neutral, one with emotionally positive, and one with emotionally negative cue words, and that each participant would start with another list.

Participants then received a booklet on which they noted a personal code. Each page of the booklet contained a space designated to write down the cue word and a short description of the corresponding event in one or two sentences, and a series of seven-point scales designated to rate the event according to its clarity, personal significance, valence, emotional arousal, and the frequency with which participants had previously thought about the event or told it to others. Participants were asked to think of events that they had personally experienced during the last 6 months, and that had lasted seconds, minutes or even hours, but not several days. They were instructed to describe unique, specific events, and not regularly recurring events or personal routines (for example, a description of a specific event would be “Last Monday when I went to the gym, I met Thomas and had a short conversation about his new apartment with him,” but not “I usually go to the gym on Monday”). Finally, they were instructed to think of a new event for each selected cue word, thus collecting a total of 16 different autobiographical events for each list. For the neutral cue words, participants were asked to think of events that did not have any emotional significance to them. For the emotionally positive and negative cue words, they were instructed to think of emotionally positive or negative events of medium intensity experienced in their daily life (thus, their daily hassles and daily uplifts), rather than of profound, intensely emotional events (as for example marriage or death of a significant other). To ensure that event descriptions would not largely vary in detail and length across events and participants, following previous research using the cue word method for the collection of autobiographical memories (Barnier et al., 2004, 2007), participants were instructed to write down a relatively brief description of one to two sentences for each event.

Depending on counterbalancing group, the experimenter first handed the list containing the neutral, emotionally positive, or emotionally negative cue words to the participants. There were no time restrictions, and the session ended when participants had recalled 16 memories for each of the three lists. It took participants between 90 and 120 min to complete the collection session. Before leaving the laboratory, participants inserted their completed booklets through a slot into a locked box. Afterwards, the booklets were collected by two independent research assistants who prepared the individualized booklets for the retrieval practice/restudy session and the first delayed memory test session.

#### Retrieval Practice/Restudy Session

The retrieval practice/restudy session took place 1 day after the collection session. At the beginning of the session, participants received via their personal code individualized booklets that were prepared before the session. For each participant, the completed booklet of the collection session was scanned, and the cue words and corresponding event descriptions of each page were cut out and stored as image files. The events were assigned alternating one by one to the retrieval practice condition or to the restudy condition, following the order in which they had been described in the collection session; the assignment of the first event was counterbalanced across participants. In this way, for each participant, half of the events of each emotional quality were retrieval practiced, and the other half restudied. For retrieval practiced events, only the image of the cue word was pasted on a single page; for restudied memories, the image of the cue word and the image of the corresponding event description were pasted on a single page. The order of retrieval practice/restudy was blocked by emotional quality, following the same order as in the collection phase. Within each emotional quality block, events were presented blocked for retrieval practice or restudy; the order of retrieval practice/restudy was counterbalanced across participants. Overall, participants completed three retrieval practice/restudy cycles, with a short break of 40 s between cycles.

Participants were instructed that for half of the events they had collected the previous day, only the cue word would be presented in the booklet, and that in this case their task would be to remember the corresponding event and describe it in the designated space beneath the cue word. For the other half of the memories, the cue word would be presented together with the corresponding event description, and in this case, their task would be to carefully read the cue word and the event description, and to copy the event description in the designated space beneath. A time slot of 40 s was allotted for restudying or retrieving one event. When time was up, participants were notified by an acoustic signal to turn the page in their booklet and continue working on the next event. The total duration of the retrieval practice/restudy session was around 100 min; at the end of the session, participants again inserted their completed booklets into the locked box.

#### First Delayed Memory Test Session

Two weeks after the retrieval practice/restudy session, participants returned to the laboratory for the first delayed memory test session. At the beginning of the session, they received their individualized test booklet via their personal code. The test booklets contained, for all 48 originally collected autobiographical events, a page showing only the corresponding cue word; the order followed the order in which the events had been described in the collection session. Participants were instructed to remember the autobiographical event they had described in response to the cue word in the first session of the experiment, and to write a description of the events in the designated space beneath the cue word. For each event, a time slot of 40 s was allotted, after which a signal tone notified participants to turn the page of the booklet and try to remember the next event. After finishing the test, participants answered a questionnaire unrelated to the study question. Then, they inserted their completed test booklets into the locked box.

#### Second Delayed Memory Test Session

Thirteen weeks after the retrieval practice/restudy session, participants received an email with a link to the second delayed memory test that was performed within an online survey environment (SoSci Survey; Leiner, 2014). Participants could access their individualized memory test via their personal code. The cue words participants had originally selected in the collection phase were presented one by one on a single page each, following the order in which the events had been described in the collection session. Participants were prompted to remember the autobiographical event they had described in response to the cue word in the first session of the experiment, and type it in the designated space beneath the cue word; they were asked to write “no memory” if they definitely could not recall the corresponding event. There was no time restriction; however, to ensure that participants would really try to recover their memories rather than only click through the test, they had to stay at the same page for at least 15 s until a button appeared through which they could progress to the next cue word. The whole test had to be completed within one session that took participants on average 27 min. After the memory test, participants answered a questionnaire unrelated to the study question.

Twenty-nine participants undertook the memory test on the same day that they had received the invitation email or on the following day, 18 participants undertook the memory test within 1 week after receiving the invitation email, and one participant did not take part in the delayed memory test. Thus, the participants’ individual retention intervals between retrieval practice/restudy and the second delayed memory test lay between 13 and maximal 14 weeks.

### Scoring

Memories were scored as correctly remembered if the event description at recall corresponded to the event description given for the corresponding cue word in the collection session. Following the scoring procedure applied by Barnier et al. (2004, 2007) exact correspondences were not required, but there had to be a clear relation between the descriptions of the events described for the same cue word at recall and in the collection session, that is, they had to contain at least some of the same information and unambiguously refer to the same event. For instance, if the original description was “my boyfriend and I were drinking wine in front of the Eiffel tower,” then “wine under Eiffel tower” was scored as correctly, but “trip to Paris” not, because the latter description does not unambiguously refer to the same event. Similarly, if the original description was “a few weeks ago I went running and discovered a nice little park,” then “I discovered a nice park” was scored as correctly, but “a sunny day and going for a walk” not, because the latter description does not clearly relate to the original description.

Two raters independently scored all recall protocols of the retrieval practice and delayed memory test sessions. Cohen’s Kappa (κ) was performed to determine consistency among raters and indicated high inter-rater reliability, κ = 0.94 (*p* < 0.001), 95% CI (0.93, 0.95). Finally, any discrepancies between raters were solved by a third rater.

## Results

**FIGURE 2:**
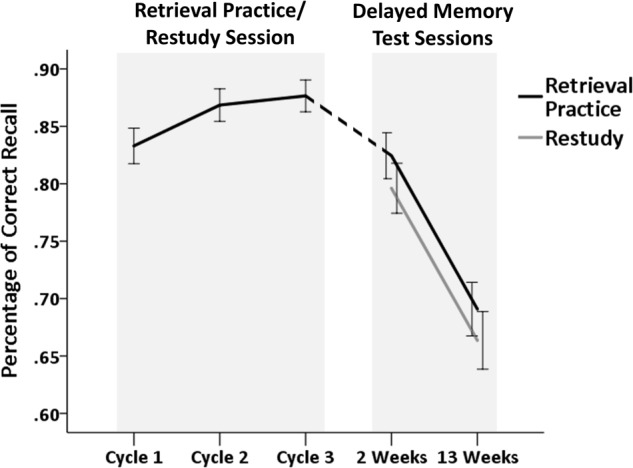
Percentage of correct recall of autobiographical events in the retrieval practice/restudy session (cycle 1, cycle 2, and cycle 3) and in the delayed memory test sessions (2 weeks, 13 weeks) as a function of the type of previous practice (retrieval practice, restudy). Error bars represent standard errors of the means.

Memory performance in the retrieval practice/restudy session (cycle 1, cycle 2, cycle 3) and in the delayed memory tests (2 weeks, 13 weeks) as a function of the type of previous practice (retrieval practice, restudy) is shown in **Figure 2** (for mean recall rates among emotion conditions, see Supplementary Table 2).

### Retrieval Practice

For the retrieval practice/restudy session, a 3 (Retrieval Practice Cycle: cycle 1, cycle 2, cycle 3) x 3 (Emotion: neutral, positive, negative) analysis of variance (ANOVA) was performed on the recall rates obtained in the three consecutive retrieval practice cycles. The analysis showed a significant main effect of Retrieval Practice Cycle, Greenhouse–Geisser corrected *F*(1.39, 65.51) = 28.25, *p* < 0.001, $\eta_{p}^{2}$ = 0.375, but no significant Retrieval Practice Cycle by Emotion interaction, Greenhouse–Geisser corrected *F*(3.05, 143.36) = 0.96, *p* = 0.413, $\eta_{p}^{2}$ = 0.020, indicating that correct recall rates, independently of emotion condition, increased throughout the three successive cycles. Pairwise comparisons, collapsed over emotion conditions, showed a significant increase in memory performance from the first (*M*_Cycle 1_ = 0.83, *SD* = 0.11) to the second retrieval practice cycle (*M*_Cycle2_ = 0.87, *SD* = 0.10), *F*(1,47) = 29.89, *p* < 0.001, $\eta_{p}^{2}$ = 0.389, and a less substantial, but significant increase from the second to the third retrieval practice cycle (*M*_Cycle3_ = 0.88, *SD* = 0.10), *F*(1,47) = 4.74, *p* = 0.035, $\eta_{p}^{2}$ = 0.092. There was also a significant main effect of Emotion, *F*(2, 94) = 3.80, *p* = 0.026, $\eta_{p}^{2}$ = 0.075. Pairwise comparisons, collapsed over retrieval practice cycles, revealed that this main effect was driven by a significant difference between recall rates for neutral (*M*_Neutral_ = 0.89, *SD =* 0.13) and positive memories (*M*_Positive_ = 0.83, *SD =* 0.15), *F*(1,47) = 7.13, *p* = 0.010, $\eta_{p}^{2}$ = 0.132, while there were no significant differences between negative (*M*_Negative_ = 0.86, *SD =* 0.13) and neutral, *F*(1,47) = 2.60, *p* = 0.114, $\eta_{p}^{2}$ = 0.052, or positive memories, *F*(1,47) = 1.45, *p* = 0.235, $\eta_{p}^{2}$ = 0.030.

### Delayed Memory Tests

One participant dropped out before the second delayed memory test session. Therefore, all analyses of correct recall rates in the delayed memory tests are based on the remaining sample of 47 participants (45 females, *M*_Age_ = 21.3, *SD* = 5.6). A 2 (Retention Interval: 2 weeks, 13 weeks) × 2 (Type of Practice: retrieval practice, restudy) × 3 (Emotion: neutral, positive, negative) repeated measure ANOVA revealed a significant main effect of Retention Interval, *F*(1, 46) = 118.53, *p* < 0.001, $\eta_{p}^{2}$ = 0.720, indicating a significant decrease in correct recall rates after a retention interval of 13 weeks compared to a retention interval of 2 weeks (*M*_2 weeks_ = 0.82, *SD =* 0.12 vs. *M*_13 weeks_ = 0.68, *SD =* 0.15, collapsed over emotion and practice conditions). There was no main effect of Emotion, *F*(2, 92) = 2.60, *p* = 0.080, $\eta_{p}^{2}$ = 0.054, nor a significant Emotion by Retention Interval interaction, *F*(2, 92) = 2.41, *p* = 0.095, $\eta_{p}^{2}$ = 0.050, indicating that correct recall rates did not significantly differ for neutral, positive or negative memories. The analysis showed no significant main effect for Type of Practice, *F*(1, 46) = 2.53, *p* = 0.118, $\eta_{p}^{2}$ = 0.052, nor a significant Type of Practice by Retention Interval interaction, *F*(1, 46) = 0.02, *p* = 0.904, $\eta_{p}^{2}$ < 0.001. Hence, recall rates for repeatedly retrieved memories did not significantly differ from recall rates for repeatedly restudied memories, neither after a retention interval of 2 weeks (*M*_RetrievalPractice_ = 0.82, *SD =* 0.14 vs. *M*_Restudy_ = 0.80, *SD =* 0.15, collapsed over emotion conditions), nor after a retention interval of 13 weeks (*M*_RetrievalPractice_ = 0.69, *SD =* 0.16 vs. *M*_Restudy_ *= 0.*66, *SD =* 0.17, collapsed over emotion conditions) (see **Figure 2**). There was also no significant Type of Practice by Emotion interaction, *F*(2,92) = 0.85, *p* = 0.431, $\eta_{p}^{2}$ = 0.018, nor a significant three-way interaction between Retention Interval, Type of Practice, and Emotion, *F*(2,92) = 2.49, *p* = 0.089, $\eta_{p}^{2}$ = 0.051.

## Discussion

The present research addressed the question whether the testing effect, that is, the finding that active retrieval from memory benefits long-term retention more than repeatedly restudying, extends to autobiographical memory. For this purpose, participants repeatedly retrieved or repeatedly restudied personally experienced autobiographical events that they had experienced within the last 6 month. In a delayed memory test 2 weeks after the retrieval practice/restudy session, no significant difference emerged between recall rates for previously repeatedly retrieved and previously repeatedly restudied autobiographical events. Importantly, this pattern persisted even after a long retention interval of over 3 months; while overall recall performance significantly decreased from the medium to the long retention interval, this decline was not any different for previously retrieved than for previously restudied events, thus ruling out the possibility that the benefits of retrieval practice in autobiographical memory might only emerge after comparably long retention intervals (Kornell et al., 2011). Thus, it seems that the typical testing effect commonly found for non-personal information does not extend to autobiographical memory representations of personally experienced events.

There are several possible explanations for this finding. Autobiographical memory is typically conceived as highly associatively organized (Conway and Pleydell-Pearce, 2000; Conway, 2005). This unique characteristics of autobiographical memory representations may at the one hand entail a comparably stronger benefit of simple re-exposure in the form of restudying, as it is well documented in previous research that memory performance is increased for highly associatively organized information (for a review, see Bower, 1970). On the other hand, the memory boost received from retrieval practice is often explained by the enhancement and formation of new associative links between memory traces following successful retrieval (e.g., Carpenter, 2011), and may thus be less pronounced for autobiographical information which is inherently organized in a highly associative network. Both mechanisms, either on their own or in conjunction, can account for the present finding that repeatedly retrieving memories for autobiographical events does not benefit long-term retention for those memories any more than repeatedly restudying them. Indeed, similar theoretical assertions have been made for the finding that the testing effect is less pronounced or disappears when inherently relationally organized learning material is concerned (van Gog and Sweller, 2015). Furthermore, it may also be that the decreased effectiveness of testing compared to restudying in autobiographical memory is attributable to other characteristics of autobiographical memories such as, for instance, high self-relevance (Conway and Pleydell-Pearce, 2000; Conway, 2005). Memory performance for self-relevant information is increased as well (for a meta-analysis, see Symons and Johnson, 1997), and high self-relevance may lead to an enhanced associative processing during restudying due to increased interest in the restudied contents (for a review, see, e.g., Schiefele, 2001). Indeed, an interesting avenue for future research would be to determine the specific mechanisms underlying the decreased testing effect in autobiographical memory by comparing, for instance, the effect of testing on memories for autobiographical and matched non-autobiographical events.

The present findings are also relevant for the literature on autobiographic memory rehearsal. In previous literature, rehearsal of personal events in social communication and various types of private autobiographical memory rehearsal have been distinguished (e.g., Walker et al., 2009). However, since typically rehearsal types have been examined that involve retrieving personally experienced events from memory, less is known about the relative significance of retrieving autobiographical events compared to simple re-exposure to those events. From an applied perspective, the latter may occur when listening to rehearsals of socially shared personally experienced events in conversations, but also individually, for example through re-exposure to autobiographical events stored via different media (e.g., diaries, videos, social media postings; see Wang et al., 2017). In line with previous results demonstrating that retrieval by talking about socially shared personally experienced events benefits long-term retention similarly to re-exposure by listening to the rehearsal of those events (Stone et al., 2013), the present results show that repeatedly retrieving autobiographical events does not benefit long-term retention significantly more than repeated re-exposure to those events. This indicates that the pattern reported for memory rehearsal in social contexts may also extend to forms of individual rehearsal of and re-exposure to autobiographical events.

It is important to note that the autobiographical memory paradigm (Barnier et al., 2004) that was adapted in the present study to examine post-encoding effects in autobiographical memory involves retrieval components in the initial collection phase where individual autobiographical memories are collected in response to cue words. In fact, alternative ways of collecting autobiographical memories that do not involve initial retrieval by participants (e.g., recording the daily life of participants) seem hardly feasible for both economical and ethical reasons. Thus, the autobiographical memories in the restudy condition had been at least one time retrieved before memory was measured in the 2-weeks-delayed memory test. However, this does not necessarily reflect a problem since it seems to be a natural characteristic of autobiographical memories that they are retrieved from time to time, as also shown in the present study by the ratings of frequency of previous retrieval of the collected autobiographical memories (mean of 3.3 on a scale ranging from 1 = not at all to 7 = very often). In fact, it may be that previous retrieval is one of the reasons why testing may be less effective than restudying in autobiographical memory compared to other types of memories.

One potential caveat regarding the interpretation of the memory results in the 13-weeks-delayed memory test may be that memory performance may have been influenced by the fact that both tested and restudied memories had already been tested once in the 2-weeks delayed memory test. More precisely, according to distribution-based explanations of the testing effect (Halamish and Bjork, 2011; Kornell et al., 2011), it may be that actually existing differences in memory strength between tested and restudied autobiographical memories are not detectable at retention intervals of 2 weeks, but only at longer retention intervals because the memory strengths of both types of memories are still above retrieval thresholds after 2 weeks. As both types of memories were tested in the 2-weeks-delayed memory test, both tested and restudied memories may have received a (comparable) additional boost in memory strength so that actually existing differences were still not detectable at the time of the 13-weeks delayed memory test. However, such a possibility would presuppose relatively slow forgetting rates due to the relatively long delay of the final test (13 weeks), which seems unlikely given that a comparatively strong memory decay was observed in the present study between the 2-week-delayed and the 13-weeks-delayed memory tests. Still, it is important to note that future research should examine potential effects of moderating variables such as the number or the spacing of retrieval practice in order to investigate the generalizability of the present findings. Thus, from a broader perspective, the present research represents an important starting point for future research on the effects of testing on long-term retention of autobiographical memories.

The fact that we did not observe any difference between a testing and a restudy condition indicates that the testing effect (in the sense of a comparison between testing and restudy) seems not to occur in autobiographical memory. Still, it may be an interesting question for future research to compare the effects of re-exposure (testing or restudy) to a no-treatment control condition in autobiographical memory. Furthermore, future research is also necessary to evaluate whether the present findings generalize beyond specific circumstances of the present experiment. First, as the present sample consisted mainly of female undergraduate students, future research should investigate the generalizability of the findings across gender and age groups. Second, as the second delayed memory test took place in an online setting, future research should examine, whether similar long-term effects are found when testing in a laboratory setting.

The present study also contributes to another line of research addressing the effects of repeated testing. In the retrieval practice/restudy session, the amount of correctly recalled autobiographical events significantly increased throughout the three retrieval cycles, a phenomenon commonly referred to as “hypermnesia,” that is, the improvement of memory performance across varying retention intervals (for a review see Payne, 1987; Erdelyi, 2010). Concerning autobiographical memories, previous research has demonstrated hypermnesia to occur across various retellings of one autobiographical event (Bluck et al., 1999). The recall pattern observed across retrieval cycles in the retrieval practice/restudy session of the current study indicates that the hypermnesic effect also extends to autobiographical memories of multiple events retrieved in response to external cues. This observation is in line with previous interpretations of hypermnesia emphasizing the role of imagery encoded study material (e.g., Erdelyi, 2010), as autobiographical memories have been demonstrated to be highly imagery (Brewer, 1992).

Interestingly, in the present study, emotional memories were not better remembered than neutral memories. By contrast, in the retrieval practice phase, neutral memories were even significantly better recalled than positive memories. At first glance, this seems to be at odds with the common finding that memory is enhanced for emotionally significant compared to neutral events (for a review, see Reisberg and Heuer, 2004). However, previous studies have shown that the emotional enhancement effect disappears when controlling for memory-enhancing cognitive factors such as the distinctiveness or relatedness of the stimuli (Schmidt and Saari, 2007; Talmi et al., 2007). Talmi et al. (2007) offer two possible explanations for these findings. First, because emotional stimuli are usually inherently more distinct, related, or attention grabbing than neutral stimuli, the typically observed emotional enhancement effect might at least be partially mediated by these cognitive factors. Alternatively, the manipulation of these factors may have simply raised memory performance for neutral stimuli to the same level as for emotional stimuli, regardless of possibly different underlying processes for emotional and neutral stimuli. Thus, in the current study, the collected neutral autobiographical events may have exhibited features that made them somehow cognitively distinct, especially as most autobiographical memories are typically emotionally significant (Brewer, 1988). More precisely, as participants were specifically instructed to think of neutral autobiographical events, they may have selected events that stood out compared to other neutral events (e.g., events that involved the breaking of a routine or low-frequent events; Brewer, 1988), which in turn may have led to equal memory performance for neutral and emotionally significant memories. Additionally, in the present study, we focused on moderately emotionally intense autobiographical events. As many explanations of the emotional enhancement effect emphasize the central role of emotional arousal (for a review, see McGaugh, 2004), it may also be that the events collected in the present study were not emotionally arousing enough for the emotional enhancement effect to emerge, especially if neutral events also exhibited distinct, memory-supporting features. Thus, concerning the effect of retrieval practice on autobiographical memories, it may well be that the pattern of results would differ for intensely emotionally arousing autobiographical events, an interesting question that should be addressed in future research.

## Conclusion

Retrieving events from memory in a test has been demonstrated to provide a powerful boost for long-term retention of those events, even compared to repeatedly restudying the event. While the benefits received from testing have been shown across a variety of stimuli material and recall tasks, one important memory system has not been addressed in previous research: autobiographical memory. In the present study, the testing effect paradigm was extended to autobiographical memories of personally experienced events. Across two delayed memory tests with retention intervals of up to 3 months, no significant memory benefit emerged for previously repeatedly retrieved compared to previously repeatedly restudied autobiographical events. Thus, it seems that the testing effect does not persist in autobiographical memory.

## Author Contributions

KE prepared the draft manuscript and CK provided critical revisions. All authors contributed to the study design, analyzed and interpreted the data, and approved the final version of the manuscript for submission.

## Conflict of Interest Statement

The authors declare that the research was conducted in the absence of any commercial or financial relationships that could be construed as a potential conflict of interest.
