# Supplementary material for: Testing Memories of Personally Experienced Events: The Testing Effect Seems Not to Persist in Autobiographical Memory
Source: Front Psychol. 2018 May 24;9:810. doi: 10.3389/fpsyg.2018.00810 (PMC5976790; doi:10.3389/fpsyg.2018.00810)
Supplement: Supplementary file 1 [file Table_1.PDF]

Supplementary Material

Testing Memories of Personally Experienced Events: the Testing Effect Seems Not to Persist in Autobiographical Memory

Kathrin J. Emmerdinger*, Christof Kuhbandner

*** Correspondence:** Corresponding Author: kathrin.emmerdinger@ur.de

Supplementary Tables

Supplementary Table 1.

Neutral, positive and negative cue word lists used in the experiment (original German cue words, English translations in brackets).

Cue Word Lists					
Neutral		Positive		Negative	
ausleihen *(to borrow/to lend)*	ordnen *(to arrange/to organize)*	Erfolg *(success)*	herzlich *(cordial)*	Streit *(quarrel)*	peinlich *(embarrassing)*
Mensa *(canteen)*	Frage *(question)*	beschwingt *(elated)*	Sport *(sports)*	Abschied *(farewell)*	krank *(sick)*
Uhr *(clock/watch)*	Jacke *(jacket)*	sonnig *(sunny)*	Freundschaft *(friendship)*	Zeitnot *(shortage of time)*	fürchten *(fear)*
Fenster *(window)*	Termin *(appointment)*	helfen *(to help)*	lecker *(delicious)*	frieren *(to be cold)*	tragisch *(tragic)*
anmelden *(to register)*	Boden *(floor)*	Feier *(celebration/ party)*	Natur *(nature)*	nervös *(nervous)*	versagen *(to fail)*
Haushalt *(housekeeping)*	drucken *(to print)*	Glück *(happiness/ luck)*	tanzen *(to dance)*	einsam *(lonely)*	Lüge *(lie)*

Schere<br>*(scissors)*	Frisur<br>*(hairstyle)*	Ausflug<br>*(excursion)*	gesellig<br>*(convivial)*	unfair<br>*(unfair)*	Pech<br>*(misfortune)*
aufladen<br>*(to charge)*	Tisch<br>*(table)*	spielen<br>*(to play)*	Tier<br>*(animal)*	Ärger<br>*(anger)*	Übelkeit<br>*(nausea)*
Stift<br>*(pen)*	gehen<br>*(to walk)*	entspannt<br>*(relaxed)*	lachen<br>*(to laugh)*	verirren<br>*(to get lost)*	gemein<br>*(mean)*
Schuh<br>*(shoe)*	egal<br>*(indifferent)*	Kompliment<br>*(compliment)*	kreativ<br>*(creative)*	Fehler<br>*(mistake)*	verpassen<br>*(to miss [e.g. the train])*
öffnen<br>*(to open)*	Werkzeug<br>*(tool)*	unterhaltsam<br>*(entertaining)*	Belohnung<br>*(reward)*	verlieren<br>*(to loose)*	anekeln<br>*(to disgust)*
einkaufen<br>*(to buy/to shop)*	aufheben<br>*(to pick up)*	Geschenk<br>*(present)*	energiegeladen<br>*(energetic)*	traurig<br>*(sad)*	Panne<br>*(mishap/ breakdown)*
Lesesaal<br>*(reading room)*	Brief<br>*(letter)*	Familie<br>*(family)*	umarmen<br>*(to embrace)*	Absage<br>*(rejection)*	entsetzt<br>*(appalled)*
E-Mail<br>*(e-mail)*	mitgeben<br>*(to give to take with them)*	freuen<br>*(to rejoice)*	Reiseerlebnis<br>*(travel experience)*	Gestank<br>*(stench)*	allergisch<br>*(allergic)*
Lampe<br>*(lamp)*	Wohnung<br>*(apartment)*	begeistert<br>*(enthusiastic)*	verliebt<br>*(enamoured)*	Problem<br>*(problem)*	Vorwurf<br>*(reproach)*
klingeln<br>*(to ring/to ring the bell)*	fahren<br>*(to drive/to go [by vehicle])*	Musik<br>*(music)*	Gespräch<br>*(conversation)*	verletzen<br>*(to injure)*	machtlos<br>*(powerless)*
Tasche<br>*(bag)*	Eimer<br>*(bucket)*	Kind<br>*(child)*	warm<br>*(warm)*	Sorge<br>*(worry)*	schlaflos<br>*(sleepless)*
