# Supplementary material for: Testing Memories of Personally Experienced Events: The Testing Effect Seems Not to Persist in Autobiographical Memory
Source: Front Psychol. 2018 May 24;9:810. doi: 10.3389/fpsyg.2018.00810 (PMC5976790; doi:10.3389/fpsyg.2018.00810)
Supplement: Supplementary file 2 [file Table_2.PDF]

Supplementary Material

Testing Memories of Personally Experienced Events: the Testing Effect Seems Not to Persist in Autobiographical Memory

Kathrin J. Emmerdinger*, Christof Kuhbandner

*** Correspondence:** Corresponding Author: kathrin.emmerdinger@ur.de

Supplementary Tables

Supplementary Table 2.

Mean recall rates for autobiographical events during retrieval practice (cycle 1, cycle 2, cycle 3) as a function of emotion condition (neutral, positive, negative) and mean recall rates for autobiographical events in the delayed memory tests (2 weeks, 13 weeks) as a function of emotion condition (neutral, positive, negative) and the type of previous practice (retrieval practice, restudy).

			Emotion Condition					
			Neutral		Positive		Negative	
			M	*SD*	*M*	*SD*	*M*	*SD*
Retrieval Practice	Cycle 1		.87	.15	.79	.17	.83	.14
	Cycle 2		.90	.13	.84	.15	.86	.14
	Cycle 3		.90	.13	.85	.14	.88	.12
Delayed Memory Tests	2 Weeks	Retrieval Practice	.86	.18	.81	.17	.82	.17
		Restudy	.80	.18	.80	.19	.81	.17
	13 Weeks	Retrieval Practice	.73	.20	.67	.23	.68	.22
		Restudy	.70	.21	.68	.21	.61	.22
