# Supplementary material for: Testing Memories of Personally Experienced Events: The Testing Effect Seems Not to Persist in Autobiographical Memory
Source: Front Psychol. 2018 May 24;9:810. doi: 10.3389/fpsyg.2018.00810 (PMC5976790; doi:10.3389/fpsyg.2018.00810)
Supplement: Supplementary file 3 [file Data_Sheet_1.PDF]

Supplementary Material

Testing Memories of Personally Experienced Events: the Testing Effect Seems Not to Persist in Autobiographical Memory

Kathrin J. Emmerdinger*, Christof Kuhbandner

*** Correspondence:** Corresponding Author: kathrin.emmerdinger@ur.de

Supplementary Data

Further Analysis of Memory Characteristic Ratings

1. Descriptive Statistics

Supplementary Table 3 shows participants' mean ratings for the characteristics (clarity, personal relevance, emotional valence, emotional arousal and frequency of previous retrieval) of the autobiographical memories they described in response to neutral, negative or positive cue words as a function of the assigned practice condition (retrieval practice, restudy).

Supplementary Table 3

Participants' mean ratings for the characteristics of the autobiographical memories described in response to neutral, positive, and negative cue words as a function of assigned practice condition (retrieval practice, restudy).

		Emotional Quality of Cue Word					
		Neutral		Positive		Negative	
Ratings of Memory Characteristics	Practice Condition	*M*	*SD*	*M*	*SD*	*M*	*SD*
Clarity	Retrieval Practice	5.05	1.09	5.98	0.60	5.72	0.55
	Restudy	5.08	0.98	5.97	0.69	5.79	0.54
Personal Relevance	Retrieval Practice	2.50	1.00	4.82	0.97	4.46	0.87
	Restudy	2.51	0.90	4.85	0.91	4.40	1.10
Emotional Valence	Retrieval Practice	4.07	0.30	6.00	0.53	2.25	0.43
	Restudy	4.08	0.28	6.02	0.48	2.24	0.50
Emotional Arousal	Retrieval Practice	2.24	1.08	4.71	0.91	4.80	0.81
	Restudy	2.21	1.01	4.71	0.86	4.81	0.96
Frequency of Previous Retrieval	Retrieval Practice	1.98	0.73	3.96	1.04	3.80	0.99
	Restudy	2.01	0.67	4.02	0.90	3.93	0.97

Note. Participants rated their memories in terms of clarity (1 = not clear at all, 7 = very clear), personal relevance (1 = not relevant at all, 7 = very relevant), emotional valence (1 = very negative, 7 = very positive), emotional arousal (1 = not at all emotionally arousing, 7 = very emotionally arousing), and the frequency with which they had previously thought of this event or told others about it (1 = not at all, 7 = very often).

2. Inferential Statistics

For each memory characteristic, a 3 (Emotion: neutral, positive, negative) × 2 (Type of Practice: retrieval practice, restudy) ANOVA was computed on participants' ratings.

2.1 Clarity

The analysis revealed a significant main effect of Emotion, Greenhouse-Geisser corrected $F(1.48, 69.68) = 44.99$, $p < .001$, $\eta_p^2 = .489$, but no significant main effect for Type of Practice, $F(1, 47) = 0.36$, $p = .553$, $\eta_p^2 = .008$, nor a significant Emotion by Type of Practice interaction, $F(2, 94) = 0.21$, $p = .814$, $\eta_p^2 = .004$, indicating that ratings of memory clarity were higher for emotional than for neutral memories, and that memories assigned to the retrieval practice condition and memories assigned to the restudy condition did not differ in terms of memory clarity.

2.2 Personal Relevance

The analysis revealed a significant main effect of Emotion, Greenhouse-Geisser corrected $F(1.75, 82.39) = 180.79$, $p < .001$, $\eta_p^2 = .794$, but no significant main effect for Type of Practice, $F(1, 47) = 0.02$, $p = .879$, $\eta_p^2 < .001$, nor a significant Emotion by Type of Practice interaction, $F(2, 94) = 0.28$, $p = .755$, $\eta_p^2 = .006$, indicating that ratings of personal relevance were higher for emotional than for neutral memories, and that memories assigned to the retrieval practice condition and memories assigned to the restudy condition did not differ in terms of personal relevance.

2.3 Emotional Valence

The analysis revealed a significant main effect of Emotion, Greenhouse-Geisser corrected $F(1.46, 68.50) = 1192.207$, $p < .001$, $\eta_p^2 = .962$, but no significant main effect for Type of Practice, $F(1, 47) = 0.01$, $p = .916$, $\eta_p^2 < .001$, nor a significant Emotion by Type of Practice interaction, $F(2, 94) = 0.05$, $p = .950$, $\eta_p^2 = .001$, indicating that memories described in response to negative cue words were indeed rated as more negative, $F(1, 47) = 935.02$, $p < .001$, $\eta_p^2 = .952$, and memories described in response to positive cue words as more positive, $F(1, 47) = 788.30$, $p < .001$, $\eta_p^2 = .944$, than memories described in response to neutral cue words, and that memories assigned to the retrieval practice condition and memories assigned to the restudy condition did not differ in terms of emotional valence.

2.4 Emotional Arousal

The analysis revealed a significant main effect of Emotion, Greenhouse-Geisser corrected $F(1.66, 78.09) = 206.52$, $p < .001$, $\eta_p^2 = .815$, but no significant main effect for Type of Practice, $F(1, 47) = 0.01$, $p = .907$, $\eta_p^2 < .001$, nor a significant Emotion by Type of Practice interaction, Greenhouse-Geisser corrected $F(1.77, 83.14) = 0.06$, $p = .925$, $\eta_p^2 = .001$, indicating that ratings of emotional arousal were higher for emotional than for neutral memories, and that memories assigned to the retrieval practice condition and memories assigned to the restudy condition did not differ in terms of emotional arousal.

2.5 Frequency of Previous Retrieval

The analysis revealed a significant main effect of Emotion, $F(2, 94) = 144.72$, $p < .001$, $\eta_p^2 = .755$, but no significant main effect for Type of Practice, $F(1, 47) = 1.71$, $p = .198$, $\eta_p^2 < .035$, nor a significant Emotion by Type of Practice interaction, $F(2, 94) = 0.32$, $p = .730$, $\eta_p^2 = .007$, indicating that participants rated the frequency with which they had previously thought of this event or told others about it as higher for emotional than for neutral memories, and that memories assigned to the

retrieval practice condition and memories assigned to the restudy condition did not differ in terms of the frequency of previous retrieval.

3. Discussion

For all rated memory characteristics, significant differences emerged between ratings for the characteristics of memories described in response to emotional cue words and ratings for memories described in response to neutral cue words. The ratings of emotional valence indicated, that emotional valence differed between emotional conditions as intended, with memories described in response to negative cue words rated as more negative, and memories described in response to positive cue words rated as more positive than memories described in response to neutral cue words. Additionally, memories described to both positive and negative cue words were rated as more emotionally arousing than memories described in response to neutral cue words. Regarding further memory characteristics, memories collected in the negative and positive conditions were also rated as slightly clearer, as more personally significant and more frequently previously retrieved than memories collected in the neutral condition, thus replicating the pattern for emotional and non-emotional memories found in previous studies (Barnier, Hung, & Conway, 2004; Barnier et al., 2007; Walker, Skowronski, Gibbons, Vogl, & Ritchie, 2009). Importantly however, memories assigned to the retrieval practice condition and memories assigned to the restudy condition did not differ in any of the rated memory characteristics.

4. References

Barnier, A., Hung, L., & Conway, M. (2004). Retrieval-induced forgetting of emotional and unemotional autobiographical memories. *Cognition & Emotion, 18*, 457–477. https://doi.org/10.1080/02699930340000392

Barnier, A. J., Conway, M. A., Mayoh, L., Speyer, J., Avizmil, O., & Harris, C. B. (2007). Directed forgetting of recently recalled autobiographical memories. *Journal of Experimental Psychology. General*, *136*, 301–322. https://doi.org/10.1037/0096-3445.136.2.301

Walker, W. R., Skowronski, J. J., Gibbons, J. A., Vogl, R. J., & Ritchie, T. D. (2009). Why people rehearse their memories: Frequency of use and relations to the intensity of emotions associated with autobiographical memories. *Memory*, *17*, 760–773. https://doi.org/10.1080/09658210903107846
